# Paper-Based Analytical Devices for Colorimetric and Luminescent Detection of Mercury in Waters: An Overview

**DOI:** 10.3390/s21227571

**Published:** 2021-11-14

**Authors:** Carlos Bendicho, Isela Lavilla, Francisco Pena-Pereira, Inmaculada de la Calle, Vanesa Romero

**Affiliations:** Centro de Investigación Mariña, Departamento de Química Analítica e Alimentaria, Campus de Vigo, Universidade de Vigo, Grupo QA2, Edificio CC Experimentais, As Lagoas, Marcosende, 36310 Vigo, Spain; isela@uvigo.es (I.L.); fjpena@uvigo.es (F.P.-P.); incalle@uvigo.es (I.d.l.C.); vromero@uvigo.es (V.R.)

**Keywords:** paper-based analytical devices, mercury, chromogenic and fluorogenic reagents, nanomaterials, water analysis

## Abstract

Lab-on-paper technologies, also known as paper-based analytical devices (PADs), have received increasing attention in the last years, and nowadays, their use has spread to virtually every application area, i.e., medical diagnostic, food safety, environmental monitoring, etc. Advantages inherent to on-field detection, which include avoiding sampling, sample preparation and conventional instrumentation in central labs, are undoubtedly driving many developments in this area. Heavy metals represent an important group of environmental pollutants that require strict controls due to the threat they pose to ecosystems and human health. In this overview, the development of PADs for Hg monitoring, which is considered the most toxic metal in the environment, is addressed. The main emphasis is placed on recognition elements (i.e., organic chromophores/fluorophores, plasmonic nanoparticles, inorganic quantum dots, carbon quantum dots, metal nanoclusters, etc.) employed to provide suitable selectivity and sensitivity. The performance of both microfluidic paper-based analytical devices and paper-based sensors using signal readout by colorimetry and luminescence will be discussed.

## 1. Introduction

Hg toxicity has been known from the age of Hippocrates (400 BC). Chronic poisoning episodes occurred until the beginning of the twentieth century as a consequence of Hg vapor absorption or due to inorganic salts. From the synthesis of organic Hg compounds in 1863 and their further application as fungicides and organomercury species have provoked a large number of poisonings [1,2]. Well-known cases concerning Hg poisoning include the consumption of seeds contaminated with this kind of fungicides (Iraq, 1971) [3] or the Minamata accident (Japan, 1953) [4] due to the intake of fish contaminated with methylmercury, which was formed by biomethylation of inorganic Hg released in the bay.

Hg can reach aquatic ecosystems through point-source discharges or atmospheric deposition. Thus, volcanic eruptions and the solubilization of rocks, soils and sediments are among the most relevant natural sources [5]. Anthropogenic sources such as small-scale gold mining, the combustion of solid fuels (coal, lignite, wood), chlor-alkali, paper, paint and pharmaceutical industries, dental implants, agriculture products (germicides, pesticides, etc.); although mostly restricted in many countries, they still contribute to increasing the Hg levels in the environment [6]. Therefore, stringent analytical controls are needed to assess the contamination of environmental samples with Hg.

Hg can be found in different environmental compartments as a variety of species; each one has different behavior, and hence, toxicological properties, bioavailability and environmental impact depend on its physicochemical forms (i.e., speciation). Thus, in natural waters, the main forms in which Hg can be present are elemental mercury (Hg^0^), inorganic mercury (Hg^2+^) and organic mercury, i.e., CH_3_-Hg^+^ and (CH_3_)_2_Hg. Biomagnification of Hg through the food chain may occur as a result of the high hydrophobicity of organic Hg species. In this way, Hg can accumulate in some fish by a factor of ca. 10^6^ in respect to the concentration levels in the aquatic environment [7].

Freshwater sources are essential for life and their scarcity can be aggravated by overexploitation of water supply systems, overuse, poor management, decreased rainfall, global warming and land use [8]. For drinking water, several regulations have established the maximum contaminant levels for Hg(II). Thus, the World Health Organization (WHO) recommends a guideline value of 6 ppb [9]. The United States Environmental Protection Agency (USEPA) fixed a maximum contaminant level of 2 ppb [10], whereas the European Directive pointed out a parametric value of 1 ppb [11].

For the determination of Hg at the (ultra)trace level, conventional instrumentation is typically used in central labs on a routine basis, such as cold vapor-atomic absorption spectrometry (CV-AAS) [12], cold vapor-atomic fluorescence spectrometry (CV-AFS) [13], electrothermal atomic absorption spectrometry (ETAAS) [14], inductively coupled plasma-mass spectrometry (ICP-MS) [15] and total reflection X-ray fluorescence (TXRF) [16]. While these techniques provide adequate sensitivity and precision, they require suitable sampling, preservation procedures, sample pretreatment and a fully controlled laboratory environment, which makes it difficult to extend their application for on-field analysis [17]. In addition, problems may arise in the sampling and sample preparation procedures prior to the determination of Hg at the (ultra)trace level by conventional analytical techniques, which can lead to systematic errors and unacceptable analytical uncertainties [7].

In recent years, several trends have emerged concerning the analytical control of environmental pollutants, such as a remarkable increase in the miniaturization, portability and greenness of analytical approaches, thus facilitating on-site measurements [18]. The latter possibility is particularly interesting, since it could allow real time measurements without the need for preservation, transport and sample storing prior to analysis by a conventional technique. Further appealing features include the possibility of performing temporally and spatially discriminated analysis and the access to remote sites so that the source of pollutants, their distribution and environmental impact can be more easily assessed [19]. 

## 2. Development of Paper-Based Analytical Devices for the Detection of Mercury

**Figure 1:**
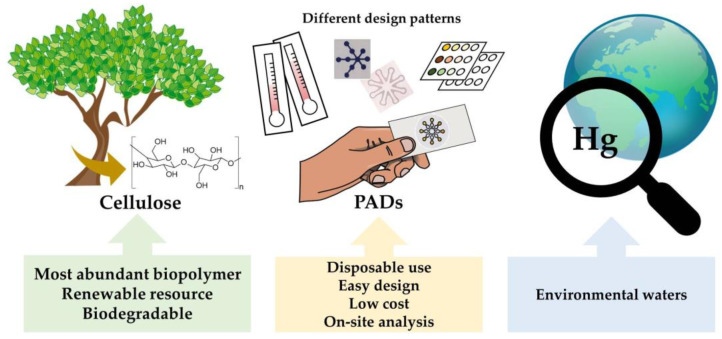
Conceptual scheme showing the application of cellulose for the detection of Hg.

Lab-on-chip (LOC) technologies have emerged as miniaturized, low cost and fast analytical approaches allowing a decrease in sample, reagents and energy consumption through the integration of typical stages of bench-scale laboratories within a single device [20]. From the standpoint of green chemistry, the use of cellulose instead of typical substrates employed in LOC systems such as polymers, silicon or glass represent a significant step forward. Cellulose-based materials have been established in the last years as efficient, versatile and universal biopolymers for the design of novel microscale analytical systems [21] (Figure 1). As compared to other scaffolds used for building sensors and microfluidic devices, cellulose is a biodegradable, biocompatible, hydrophilic and highly porous material. In addition, it possesses high capillarity, and a large variety of recognition elements can be immobilized for sensing. When used along the widespread colorimetric transduction, white color is excellent to achieve good analytical performance [22].

The so-called paper-based analytical devices (PADs) have arisen as an efficient, affordable, user-friendly, rapid, and equipment-free technology that is available to citizens. The development of PADs in areas such as clinical diagnostics, food safety and environmental monitoring, etc., as well as fabrication methods, target analytes and analytical performance, has been extensively reviewed during the last decade [23,24,25,26,27,28,29,30,31,32,33,34,35,36,37], with the scientific community showing great interest toward these appealing analytical approaches.

Under the general term ‘paper-based analytical devices’ (PADs), two systems can be distinguished, i.e., microfluidic paper-based analytical devices’ (μ-PAD), where a fluidic network is built in the paper substrate, and ‘paper-based assay devices’, also known as ‘paper-based sensors’ or ‘spot tests’, where the sample is directly deposited onto the paper surface. First systems, introduced by Whitesides for the first time [38], include different configurations, such as two-dimensional (2D), three-dimensional (3D) and distance-based devices. In these microfluidic devices, the sample and reagents are transported to the detection zone by capillarity. Second designs derive from the classical qualitative analysis tests, where the detection of inorganic cations and anions could be performed on filter paper using suitable colorimetric and fluorescent reagents [39]. In paper-based assay devices, the sample comes directly into contact with the receptor, which remains stationary on the cellulose scaffold.

**Figure 2:**
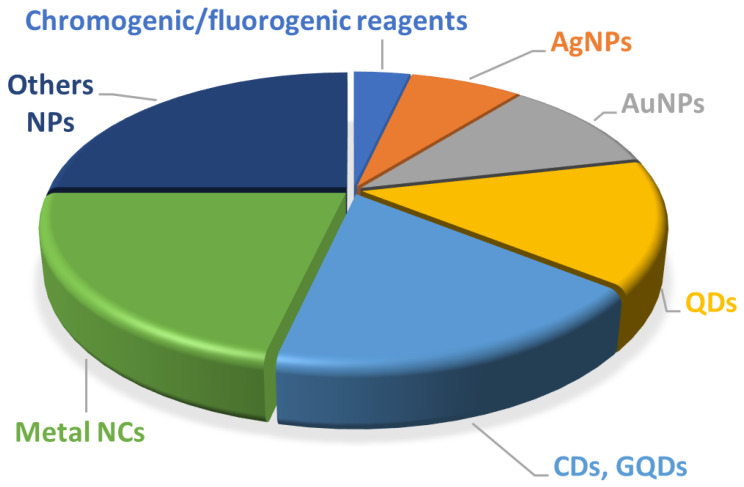
Percentage of publications related to the application of PADs with different recognition elements for sensing Hg(II) in water using colorimetry and luminescence. AgNPs, silver nanoparticles; AuNPs, gold nanoparticles; CDs, carbon dots; GQDs, graphene quantum dots; NCs, nanoclusters; NPs, nanoparticles; QDs, quantum dots.

A relevant group of environmental pollutants contributing to morbidity and mortality, especially in regions that lack of suitable analytical controls, is integrated by toxic heavy metals, which include some metals and metalloids possessing a relatively high density, such as Cd, Cu, Cr, Hg, Pb, Ni, As, Sb, etc. Unlike other pollutants, heavy metals are not biodegradable and can bioaccumulate in living beings, causing toxicity even when present at ultratrace levels in the environment. According to WHO [40], Hg is considered as one of the top 10 chemicals or groups of chemicals of major public health concern. Although several review papers have appeared in the literature dealing with applications of PADs for the detection of heavy metals [34,37], no specific one has been published related to Hg, an element for which there has been a sharp increase in applications over the last five years. Figure 2 shows the percentage of publications related to applications of μ-PADs and paper-based sensors to the detection of Hg(II) in the environmental field using colorimetric and luminescent signal readout. 

In this review, we provide an overview on the state of the art of PADs for the detection of Hg in environmental samples, their main shortcomings and future prospects.

## 3. Paper-Based Sensors Integrated with Organic Chromogenic/Fluorogenic Receptors for Hg Detection

Several chromogenic/fluorogenic reagents have been used as recognition elements for the detection of Hg(II) in both paper-based sensors [41,42,43,44,45,46,47] and μ-PADs [48,49,50,51] (Table 1). In a few cases, multiplexed systems for the detection of other metal ions have been reported [50,51]. An array of paper strips has also been designed for the detection of several metals, including Hg [52]. Environmental samples analyzed mostly include several types of waters, yet applications to biological samples, soils and creams have also been described [41,48,49]. With some exceptions where inorganic chromogenic species are involved [41,49], the most reported applications use organic chromogenic reagents for analyte recognition. 

Original concentration units of limits of detection (LODs) provided by authors are kept in the text, but these units have been converted into parts-per-billion (ppb) in Table 1 and Table 2 so as to facilitate comparisons. Those PADs providing an LOD equal or below the maximum contaminant level regulated by US EPA (i.e., 2 ppb) are marked with an asterisk in Table 1 and Table 2.

While naked eye detection is carried out in many PADs, devices related to information and communication technologies (ICTs) such as digital cameras, scanners, smartphones, etc., have been mostly used for capturing images on PADs. Further image processing is employed for measuring color intensity. LODs at the ppm level are generally reported for many applications of PADs concerning Hg detection, with the exception of approaches involving any kind of preconcentration (e.g., [43]). In a significant number of papers, LODs corresponding to the use of receptors in a solution followed by detection with a conventional instrument are provided (e.g., [45,46]).

**Figure 3:**
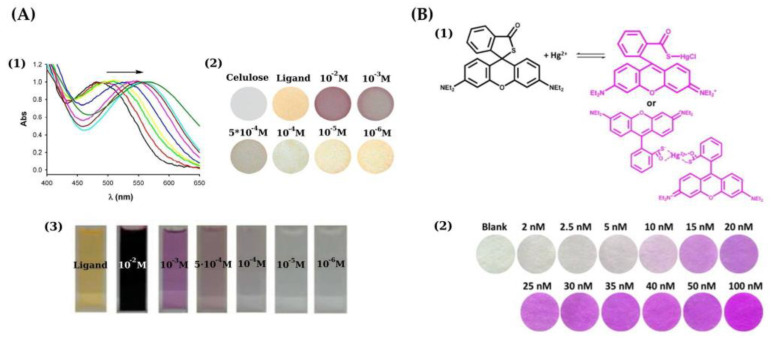
(**A**) (1) Normalized reflectance UV-Vis spectra of Hg(II) in acetonitrile upon dipping cellulose papers into solutions containing increasing concentrations; a bis(ferrocenyl) azine was used as chromogenic reagent; (2) naked-eye detection of Hg(II) using the paper-based sensor; (3) color of acetonitrile-water solutions of the chemosensor in the presence of Hg(II) at different concentration [42]. (**B**) (1) Reaction between rhodamine B thiolactone and Hg(II) to yield a purple red product; (2) paper-based sensors for Hg(II) showing the change from white to a purple-red color; a 10 nM Hg concentration can be detected with the naked eye [43]. Reproduced with permission of Elsevier [43].

Paciornik et al. [41] described the detection of Hg(II) in fish by means of cuprous iodide (CuI), which caused a yellow-orange color. An LOD of 0.007 μg/g (wet weight) was reported. Diez-Gil et al. [42] carried out the detection of Hg(II) on cellulose-based supports with the naked eye based on the reaction with bis(ferrocenyl) azine in acetonitrile-water solution (Figure 3A). An LOD about 10 mg/L was obtained.

Liu et al. [43] described the reaction between Rhodamine B thiolactone and Hg(II) giving rise to a magenta color (Figure 3B). The receptor was entrapped on porous silica matrix, and the silica layer was impregnated in a filter paper. The as-prepared membrane serves the purpose of preconcentrator and chromogenic sensing, so that sensitivity is significantly improved. The aqueous sample was passed through the filter using a vacuum aspirator at ca. a 30 mL/min flow rate. The threshold for safe levels of Hg in drinking water is around 10 nM (EPA guideline for the maximum allowable Hg level in drinking water), which could be reached with the naked eye for 200 mL of sample. Colorimetric measurements were performed with a flatbed scanner and an LOD of ca. 1.2 nM Hg was obtained.

Patil and Das [44] described a selective colorimetric and fluorometric chemosensor based on a rhodamine appended vinyl ether (RDV) probe for Hg(II) recognition. Paper strips were employed by immersing filter paper into a RDV solution. Although an LOD of 136 nM was obtained using a solution assay, the paper strip was useful for Hg detection at the ppm level (above ca. 10 ppm).

A phosphorescent iridium (III) complex-based chemosensor for Hg(II) was inkjet-printed onto filter paper. After Hg(II) recognition, the color change was observed directly with the naked eye and under UV irradiation [45]. A detection limit for the assay carried out in solution of 1.78 × 10^−8^ M was achieved.

Fluorescent calix[4]arene (L) containing four pyrene groups as binding sites was immobilized onto cellulose to build a paper-based sensor for the detection of Hg(II), Zn(II) and iodide [46]. This chemosensor is integrated by an ionophore which is responsible for ion binding and fluorophore for signal transduction. The chemosensor used in this work displays an on-off-on chelation-enhanced fluorescence (CHEF)-photoinduced electron transfer (PET) phenomena for selective recognition. In the presence of Hg(II) there is fluorescence quenching, but on the contrary, in the presence of Zn(II) and iodide, there was fluorescence enhancement. LODs in the solution were 6.4 nM (Zn), 2.9 nM (Hg) and 20.9 nM (I). Selective Hg detection required masking the interference by Zn and iodide.

A tetrahydrophenazine-based fluorophore showing a donor–acceptor–donor behavior was used as a sensor for Hg(II) [47]. Besides fluorescence sensing, this sensor can also be used as a chromogenic sensor (changes in color from yellow to blue in the presence of Hg) and a paper-based sensor. The LOD of the fluorescent sensor in solution was 8 ppm Hg at neutral pH and 3 ppm Hg at a pH in the range of 1.6–2.3.

Idros et al. [50] described a μ-PAD using three indicators (ligands) for the detection of Hg, Pb, Cr, Ni, Cu and Fe. Although the sensor has the detection capability for Cr, Fe, Cu and Ni in drinking water, an LOD of 0.1 μM was obtained for Hg, which is 20 times higher than the safe level established for this element.

Apart from paper-based sensors and μ-PADs, multi-ion analysis arrays have also been investigated. A multi-ion analysis array test strip was designed for the detection of Hg(II), Ag(I) and Cu(II) through the immobilization of five specifically responsive indicators in order to achieve tunable sensitivities [52]. This sensing approach allows the simultaneous detection of several metal ions, even when they are present as a mixture in both qualitative and semiquantitative modes. A total of 18 indicator-containing formulations were printed onto a Whatman grade no.1 paper. Images of the strip test array were taken using a flatbed scanner and processed by Adobe Photoshop software. The LODs defined here as three times the signal-to-noise ratio were 0.19, 1.69 and 1.4 μM, which were lower than their Chinese wastewater discharge standard concentrations. The array test strip displays good anti-interference capability and storage stability as well as good production reproducibility.

Besides typical cellulose substrates, the use of cellulose nanofibers has also been reported for sensing Hg. Al-Shamsi et al. [53] reported the detection of Hg(II) using time-resolved photoluminescent measurements based on 6-thienyl-lumazine (TLm) fluorophores in cellulose acetate nanofibers. Hg(II) quenched the solid-state fluorescence of TLm through different mechanisms, i.e., dynamic and static, allowing a detection at 50 pM level.

The immobilization of an infrared fluorescence protein (IFP) and its chromophore biliverdin (BV) has been applied by Gu et al. [54] for Hg(II) detection. An LOD of less than 50 nM was achieved. The IFP/BV sensor can serve as a tool for the detection of Hg in living organisms or tissues. A protein-hydrogel-based paper assay was also used for the immobilization of IFP onto paper strips for detection of Hg(II). Enrichment by multiple addition/drying steps onto the paper strip allows detection at the 20 nM level.

## 4. Paper-Based Analytical Devices Integrated with Nanomaterials as Receptors for Hg Detection

A variety of nanomaterials have been applied to build novel sensing assays for Hg, such as plasmonic nanoparticles (NPs), e.g., gold nanoparticles (AuNPs), silver nanoparticles (AgNPs), gold nanorods (AuNRs), fluorescent nanoparticles, e.g., quantum dots (QDs), carbon dots (CDs), graphene quantum dots (GQDs), metal nanoclusters (NCs), etc. [55]. Signal readout has been mainly carried out using transduction techniques such as colorimetry and luminescence, yet one application of surface-enhanced Raman scattering (SERS) has also been published.

### 4.1. Plasmonic Nanoparticles

When the light of appropriate frequency interacts with some metal nanoparticles (e.g., Au, Ag, Cu), a collective oscillation of electrons at their conduction bands occurs, which is the basis for the surface plasmon resonance (SPR) phenomenon [56]. When the dimensions of metal nanoparticles are lesser than the radiation wavelength, the phenomenon is known as ‘localized surface plasmon resonance’ (LSPR). Absorption of radiation takes place when light has the same frequency as oscillations. The localized surface plasmon resonance (LSPR) absorption bands are characteristics of the metal involved in the colloidal solution, i.e., it depends on size, shape, interparticle distance, composition of the nanoparticles and refractive index of the surrounding medium. Thus, the colors displayed by colloidal solutions of AuNPs, AgNPs and CuNPs are pink, yellow and red, respectively. More interestingly, these NPs possess much higher molar extinction coefficients as compared to chromogenic agents. The molar extinction coefficients corresponding to the LSPR absorption bands of AuNPs and AgNPs are 10^8^ and 10^10^ M^−1^ cm^−1^, respectively. Typically, the wavelength of the LSPR band is largely affected by the size and chemical environment surrounding the nanoparticles, such as the presence of capping agents, formation of amalgams, species adsorbed, etc. Noble metal nanoparticles have been widely applied for the detection of metal ions [57].

#### 4.1.1. Gold Nanoparticles

Affinity of thymine bases toward Hg(II) has driven the development of selective sensors for Hg using signal readout under different transduction principles, such as colorimetry, luminescence and electrochemistry [19]. When DNA-capped AuNPs are brought into contact with Hg(II) ions, a shift of the SPR band occurs as a result of aggregation caused by the binding between two thymine bases and Hg(II), so the colloidal solution of AuNPs changes its color from red to blue.

He et al. [58] developed a lateral flow strip for visual detection of Hg(II) which is based on AuNPs and thymine(T)-rich Hairpin DNA probes. The sensing approach relies on hairpin DNA-conjugated AuNPs and thymine-Hg(II)-thymine coordination chemistry and immune-capturing events. A red color in the test and control zones of the strips occur due to aggregation of AuNPs in the presence of Hg(II). An LOD of 0.1 nM Hg(II) can be reached in waters without interference due to other metal ions.

Based on the AuNPs thymine-Hg(II)-thymine coordination chemistry, Chen et al. [59] reported the use of single-strand DNA (ssDNA) attached to AuNPs for detecting Hg(II). After incubation for 30 min, the solution test was deposited onto a μ-PAD and the image capture was performed by a smartphone camera. An LOD of 10 ppb Hg was achieved. AuNPs aggregation causes the color to change from red to purple.

**Figure 4:**
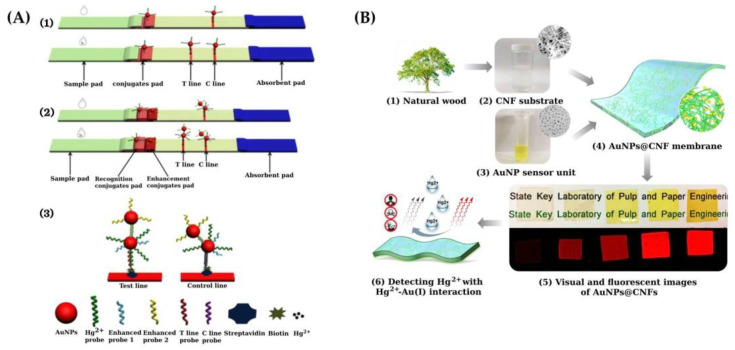
(**A**) (1) Scheme showing the fundamentals of sensing based on traditional lateral flow strips; (2) fundamentals of sensing based on signal amplified lateral flow strip; (3) detection of Hg(II) following specific recognition of T-rich ssDNA probes against Hg^2+^ resulting in the highly stable T–Hg^2+^–T complex [60]. (**B**) Scheme showing the design and sensing principle of cellulose nanofibrillated matrix-support gold nanoparticle membrane (AuNPs@CNF). (1) Starting material of natural wood. (2) CNF aqueous suspension. Inset: AFM of CNF. (3) AuNP aqueous dispersion. Inset: AFM of AuNPs. (4) Transparent AuNPs@CNF membrane. Inset: Internal microstructure of the membrane. (5) Visual images under room white light (top) and fluorescent images under UV light (bottom) of the membranes. (6) Detection of Hg^2+^ according to Hg^2+^-Au(I) interaction mechanism [62]. Figure 4A,B is reproduced with permission of Elsevier [60,62].

Zhu et al. [60] reported a novel signal-amplified lateral flow strip (SA-LFS) for Hg detection where signal amplification and sensing are carried out in one step (Figure 4A). The system is based on the specific recognition of thymine-Hg(II)-thymine using AuNPs as labeling tags. T-rich ssDNA and ssDNA are used as recognition and enhancement probes, respectively. The specificity of the T-Hg(II)-T interaction makes the test highly selective. An LOD of 0.005 ppb and 0.0015 ppb Hg were obtained for visual observation and quantitative analysis, respectively.

A signal-amplified paper-matrix-based array was developed by Yao et al. [61] for the detection of Hg(II) ions. A signal reporting ssDNA probe labeled with AuNPs was immobilized onto a nitrocellulose membrane. Color intensity due to aggregation of AuNPs in the presence of Hg(II) was enhanced by the further reduction of Ag(I) onto the surface of the AuNPs. An LOD of 0.0005 ppb Hg(II) can be achieved after signal amplification by Ag staining operation, which was 500 times better than that obtained without amplification. The paper-matrix-based array allows to perform multiple sets of duplicate assays simultaneously.

Fu et al. [62] employed a transparent cellulose nanofiber matrix-supported luminescent AuNPs as a solid-state sensing membrane for the detection of Hg(II) (Figure 4B). The method is based on the high affinity metallophilic Hg(II)–Au(I) interaction mechanism. The cellulose nanofibrillatted (CNF) matrix provided a large number of immobilizing spots to the sensor unit of AuNPs. Quenching of the AuNPs@CNF membranes fluorescence occurs as the Hg(II) concentration increases. The luminescence changes induced by Hg(II) can be observed with the naked eye even at a Hg(II) concentration as low as 0.0010 μM, much lower than the limit established by EPA (0.010 μM, 2 ppb).

A μ-PAD was developed by Shariati and Khayatian [63] using AuNPs functionalized with N,N′-bis(2-hydroxyethyl)dithiooxamide as a receptor for Hg. An LOD as low as 3 ppb could be reached, and applications to food quality control and air quality monitoring were performed.

Until now, colorimetric detection had been applied in most PADs, along with plasmonic nanoparticles. An application of SERS was reported by Yang et al. [64]. An inhibited catalytic growth of surface-enhanced Raman scattering-active AuNPs onto a hydrophobically patterned paper substrate was caused by Hg(II). 4-mercaptobenzonic acid (4-MBA) was chosen as an effective Raman reporter molecule. The Raman signal was enhanced by surface plasmonic 2-[4-(2-hydroxyethel) piperazine-1-yl]ethanesulfonic acid-stabilized gold nanostars (HEPES-AuNSs). Hg(II) caused the formation of an amalgam on the solid–liquid interface of HEPES-AuNSs. Ultimately, this caused a weak signal of 4-MBA. An LOD of 0.03 nM was achieved, and the method was applied to Hg detection in environmental waters.

#### 4.1.2. Silver Nanoparticles

Several mechanisms altering the plasmonic band (changing the color from yellow to colorless) have been proposed for sensing Hg using AgNPs as receptors in PADs [65,66,67,68,69,70,71,72,73]. These mechanisms include changes in both shape and size of AgNPls [65], amalgamation (Hg-Ag) after the reduction of Hg(II) to Hg(0), resulting in the disintegration of AgNPs [66,67,69,70,73] and complexation with capping agent and redox etching due to Hg(II) resulting in amalgamation [68,72].

A paper-based sensor was developed by Apilux et al. [65] for the detection of Hg(II) in waters using AgNPs and silver nanoplates (AgNPls). The color change of AgNPls on a paper test in the presence of Hg(II) can be monitored by the naked eye. A quantitative assay can be accomplished following image capture by a digital camera along with an image processing software to yield an LOD of 0.12 ppm. Upon the accumulation of Hg on paper through multiple applications of 2 μL, an LOD of 2 ppb can be achieved. The color change of AgNPs and AgNPls can be ascribed to changes in size and shape. A sensing mechanism based on the redox reaction between Hg(II) and AgNPls was proposed.

Meelapsom et al. [66] developed a colorimetric assay for Hg(II) based on the RGB model using a double layer μ-PAD with unmodified AgNPs and a digital camera. An LOD of 0.0001 mg/L and an RSD less than 4.1% were reached. Remarkably, three orders of magnitude for the linear range were observed.

AgNPs were employed as receptors in a micro-PAD for the detection of Hg(II) with an LOD of 3 ppb, but multiple sample depositions were needed to achieve a suitable accumulation [67].

A paper-based sensing strip was developed for the detection of Hg using glucose-capped silver nanoparticles (Glu–AgNPs) as selective receptors. A 1 μM concentration could be detected with the naked eye with the μ-PAD. A mechanism based on electrostatic interaction between anionic Glu-AgNPs and cationic Hg and also a redox etching and further amalgam formation was proposed, leading to AgNPs aggregation [68].

Ismail et al. [69] proposed a PAD for the detection of Hg(II) and ammonia, using AgNPs prepared by photosynthesis. For this purpose, the reduction of Ag(I) to Ag(0) was achieved by means of an aqueous leaf extract of *Convolvulus cneorum*. In the presence of Hg, a LSPR band shift to blue took place, providing an LOD of 5 ppb Hg.

**Figure 5:**
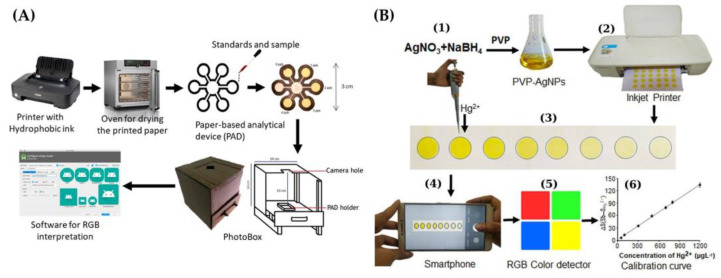
(**A**) Scheme of the PAD preparation and digital image acquisition for Hg detection using AgNPs as colorimetric probe [70]. (**B**) Scheme for inkjet-printing of PVP-AgNPs on paper; (1) synthesis of PVP-AgNPs; (2) Inkjet-printing on Whatman filter papers; (3) deposition of sample solution containing Hg(II) on the PAD; (4) image capture using a smartphone; (5) measurement of color intensity; (6) standard calibration curve [72]. Figure 5B is reproduced with permission of Elsevier [72].

Firdaus et al. [70] described a portable sensing approach for Hg(II) using AgNPs as receptors. The yellowish brown color of AgNPs became colorless in the presence of Hg(II) as result of the redox process (Figure 5A). Color processing in the PAD was carried out by the application ‘mercury detector’, available on the Google Play store. An LOD of 0.86 ppb Hg was achieved in the solution using digital image-based colorimetry.

Dey et al. [71] reported on a sensor for mercury based on a novel oxacalix[4]arene derivative, i.e., diacetamido-oxacalix[4]arene (DAOC), used for the stabilization of AgNPs (DAOC-AgNPs). The surface of the templated AgNPs was modified with rhodamine. These sensors based on oxacalix[4]arene (without or with modification with rhodamine) were seen to be selective toward MeHg^+^, providing an LOD of 1.7 nM (0.34 ppb), much lower than the EPA standard limit. The color change is explained through a mercury-induced oxidation of DAOC-AgNPs, which results in the formation of an Ag–Hg nanoalloy.

An inkjet-printed paper-based colorimetric sensor with AgNPs, along with a smartphone and RGB color detection, was developed by Shrivas et al. [72] (Figure 5B). A color change from yellow to colorless was observed in the presence of Hg(II). A reaction mechanism responsible for the color change was proposed as a result of the interaction of Hg(II) and a PVP stabilizing agent employed as a capping agent for AgNPs, and an oxidation of Ag^0^ to Ag^+^. An LOD of 10 ppb was obtained.

AgNPs synthesized through an eco-friendly procedure based on *Achillea Wilhelmsii* extracts were used to design a colorimetric paper-based sensor for monitoring Hg. A smartphone was employed for capturing images. A change in color from brown to colorless was achieved upon the addition of Hg(II) to the sensor. An LOD of 28 × 10^−9^ M Hg in aqueous solution and 0.3 × 10^−6^ M in the coated paper substrate were obtained [73].

### 4.2. Fluorescent Nanoparticles

A wide variety of luminescent nanomaterials have been employed as recognition elements and signaling agents in PADs. In most contributions, conventional instrumentation for performing luminescent measurements has been applied in conjunction with nanostructured materials, yet color measurements of the fluorescence emitted on PADs have been accomplished by capturing images of the radiation reflected and further processing. Among these nanomaterials, inorganic quantum dots (QDs) [74], carbon quantum dots (CQDs), graphene quantum dots (GQDs) [75,76] and metal nanoclusters (NCs) [77] have found applications for the detection of heavy metals, including Hg. The benefits of these nanomaterials in comparison with their organic counterparts rely on their unique optical properties, including much higher luminescent quantum yields and better photostability. Selectivity is commonly conferred by suitable surface functionalization.

#### 4.2.1. Quantum Dots

Quantum dots (QDs) are nanocrystals with a size of typically less than 10 nm that are made of different semiconductor materials and possess excellent photoluminescent characteristics [74].

**Figure 6:**
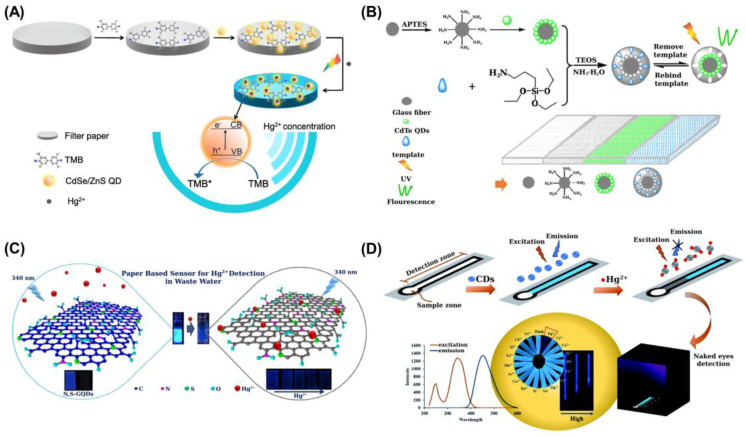
(**A**) Scheme of PAD based on the enhanced photocatalytic activity displayed by CdSe/ZnS QDs on TMB oxidation under visible light in the presence of Hg(II) [78]; (**B**) preparation of an ion-imprinted polymer and grafting onto glass fiber paper to build a 3D origami μ-PAD (Paper@QDs@IIP) [80]; (**C**) scheme showing the detection of Hg(II) based on turn-off fluorescence of N,S-GQDs; the N atoms enhance the fluorescence quantum yield while S atoms serve as the active sites for Hg(II) coordination [81]; (**D**) distance-based micro-PAD for the detection of Hg(II) using nitrogen-doped CDs as fluorescent probes [85]. Figure 6A-D is reproduced with permission of Elsevier [78,80,81] and the Royal Society of Chemistry [85].

Several PADs have been reported based on the fluorescent properties of QDs but also on their catalytic activity for oxidation of some chromogenic reagents such as 3,3,5,5-tetramethylbenzidine (TMB). Thus, CdSe/ZnS QDs exhibited enhanced catalytic activity in the oxidation of 3,3,5,5-tetramethylbenzidine (TMB) under visible light in the presence of Hg(II) [78]. The intensified blue color obtained as a result of this process can be detected with the naked eye or by the image capture using a smartphone, along with an image-processing software (Figure 6A). A paper-based sensor was built to perform the assay. An LOD of 0.09 μM Hg was achieved. The method was quite selective and was applied to real samples, such as tap water and cosmetics. The main drawback was the limited LOD (ca. 18 ppb) in comparison with well-established techniques such as cold vapor coupled with AAS, AFS and ICP-MS.

A fluorescent sensor was described by Guo et al. [79], based on MoS_2_ QDs functionalized with boronic acid for label-free detection of Hg(II) in environmental waters. Fluorescence quenching caused by Hg(II) can be observed with the naked eye under UV lamp irradiation. An LOD of 1.8 nM can be achieved. A mechanism of the Hg(II) ion-promoted transmetalation reaction of aryl boronic acid was proposed.

The ion-imprinting technique displays great potential for increasing the selectivity of recognition events in sensing template ions (Figure 6B) [80]. A novel three-dimensional (3D) origami ion-imprinted polymers (IIPs) μ-PAD for the multiplexed detection of Cu(II) and Hg(II) through the combination of microfluidic and imprinting technology was described. For this, CdTe QDs were grafted on the surface of glass fiber paper. The complex formation between Hg, Cu IIPs and CdTe QDs caused the fluorescence quenching of CdTe QDs. Three-dimensional origami μ-PADs with a ‘Y’ type channel were built. Good and reproducible results were obtained for the analysis of lake water and seawater. The LODs were 0.056 and 0.035 ppb for Hg and Cu, respectively. A main concern related to the use of inorganic QDs as recognition elements in PADs is the presence of Cd (a very toxic metal) within their composition.

#### 4.2.2. Carbon Quantum Dots and Graphene Quantum Dots

Carbon quantum dots (CQDs), also known as carbon dots (CDs), are spherical nanoparticles with a typical size of less than 10 nm, and similarly to QDs, they display strong luminescent properties. In order to improve their luminescent quantum yields, doping is usually carried out, i.e., by introducing N and S in their composition. Unlike CDs, GQDs are also strongly luminescent disks (nanosheets) of graphene with a size of 2–20 nm [74,75]

N,S-doped GQDs integrated in a paper strip have been used for the detection of Hg in wastewater (Figure 6C). Fluorescence quenching occurs in the presence of Hg as a result of its interaction with S atoms. Some sample pretreatment was required to avoid matrix effects such as filtration and solid-phase extraction. The fluorescence color was captured with a digital camera following UV irradiation [81].

A sponge cellulose fluorescence spherical (CS-CDs) was synthesized from carboxylated cellulose spheres (CCS) and citric acid (CA) and polyethylenimine (PEI) as carbon source and nitrogen doping reagents, respectively, to form CDs. Due to their porous structure, these sensors can detect ions rapidly. The detection of Cu(II) and Hg(II) was carried out under both natural light and UV light by recording color changes with a digital and mobile cameras. Selective detection of Hg(II) with an LOD of 26 nM was achieved, and its application to the analysis of tap and river water was performed. Unlike most of the PADs reported for Hg(II) sensing, which are suitable for single use only, this approach allows reusability and easy transport for on-site analysis [82]. Particularly, CS−CDs were recyclable/reusable up to six cycles by EDTA treatment.

Patir et al. [83] developed an assay for the detection of Hg(II) and Cu(II) on cellulose substrates following an on–off–on strategy. For this, N-doped CDs were synthesized from urea EDTA. The LOD obtained with the PAD for Hg was 0.1 μM. Fluorescence quenching occurs with both metal ions, but the quenched fluorescence is recovered (on) when ascorbic acid (for Hg) and citrate (for Cu) are present, and hence, both metal ions can be quantified.

Dual-colored CDs were applied by Wang et al. [84] to build a ratiometric assay for detecting Hg with the naked eye. The mechanism for this assay was based on quenching caused by Hg due to the aggregation of blue CDs, whereas the unaffected red fluorescence allowed its use as an internal reference.

A fluorescent distance-based paper device, coupled with an evaporating preconcentration system, was reported for detecting Hg(II) in water [85] (Figure 6D). For this purpose, nitrogen-doped carbon dots (NCDs) were used as fluorescent probes following a turn-off process. The heating preconcentration improved the LOD by a factor of 100 times. The LOD, detected with the naked eye under UV light, was 5 ppb Hg(II), which was low enough to establish the quality of drinking water according to the maximum contamination level regulated by the WHO.

NCDs were employed on filter paper as probes to build a fluorescent assay for Hg(II). The fluorescence of NCDs was effectively quenched by Hg(II). A smartphone was applied for capturing images of the paper chip under UV LED irradiation [86]. An LOD of 1.07 × 10^−8^ M was achieved. A non-radiative electron-transfer process from the excited states to the d orbital of Hg and the combination between Hg and N on the surface of CDs to form a non-fluorescent complex were proposed to explain the fluorescence quenching caused by Hg.

**Figure 7:**
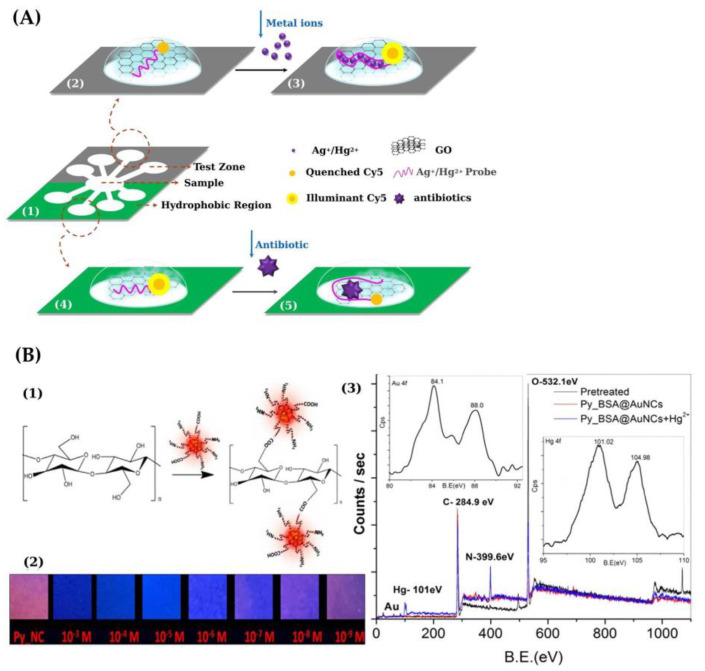
(**A**) Scheme showing a μ-PAD for multiplex detection of chemical contaminants using ssDNA-functionalized GO sensors. (1) Design of the μ-PAD. (2) and (3) show the metal ions detection mechanism based on the interaction among GO, ssDNA and heavy metals; quenching of the fluorescence occurred when Cy5-labeled ssDNA was adsorbed on the GO surface (2, fluorescence OFF). In the presence of the metal ions, ssDNA spontaneously released from the GO surface yielding fluorescence recovery (3, fluorescence ON); (4) and (5) show the principle of antibiotic detection based on the interaction between GO, ssDNA and antibiotics; the fluorescence was partly quenched when Cy5-labeled ssDNA was adsorbed with low GO concentration (4, fluorescence ON); in the presence of the aminoglycoside antibiotic, the antibiotic-probe duplex increased the bind effect between the duplex and GO surface through amide coupling, yielding a decrease in fluorescence intensity (5, fluorescence OFF) [87]. (**B**) (1) Schematic diagram showing the preparation of a paper-based sensor with Py_BSA-AuNCs as receptors; (2) fluorescence color changes observed on test paper strips with Py_BSA-AuNCs following interaction with Hg(II) at different concentration (1 mM–1 nM) under UV light at 365 nm; (3) XPS spectrum of Py_BSA-AuNCs before and after addition of Hg(II) (Inset showing the XPS spectrum of the Au 4f band and Hg 4f band) [88]. Figure 7A,B is reproduced with permission of Elsevier [87,88].

A μ-PAD was described using fluorescence labeled single-stranded DNA (ssDNA) functionalized graphene oxide (GO) for the multiplexed detection of Hg(II), Ag(I) and aminoglycoside antibiotics [87] (Figure 7A). An LOD of 121 nM was achieved for Hg.

#### 4.2.3. Metal Nanoclusters

Metal nanoclusters (NCs) made of Au, Ag, Cu, etc., with a size of less than 2 nm, does do not undergo the SPR effect, unlike metal nanoparticles (NPs), but they possess strong luminescence [77].

Bothra et al. [88] developed a nanosensor based on pyridoxal conjugated red fluorescent bovine serum albumin (BSA)-Au nanoclusters for the nanomolar detection of Hg(II) (Figure 7B). For on-site detection, a paper-based sensor was built via covalent anchoring of the nanosensor on cellulose paper. In the presence of Hg(II), the fluorescence of AuNCs was selectively quenched and the red fluorescent color observed under UV light changed to blue. An LOD of 31.9 nM Hg(II) was achieved. On-site monitoring of Hg(II) using cost-effective paper strips is feasible.

A ratiometric fluorescence probe based on Tb(III)/BSA-gold nanoclusters (AuNCs) conjugates was developed for the detection of Hg(II) [89]. In this probe, BSA-AuNCs served the purpose of signaling, whereas Tb(III) was used as the build-in reference. The probe is highly selective toward Hg(II) when present along other common environmentally and biologically relevant metal ions. An LOD of 1 nM can be achieved. A paper-based visual sensor was prepared by dripping the probe onto filter paper and underwent UV illumination with a hand-held UV lamp, which allowed for the observation with the naked eye of a Hg concentration as low as 0.1 μM.

### 4.3. Other Nanoreceptors

Curcumin nanoparticles (CURNPs) were shown to selectively respond to Hg(II). A change in color from yellow to light yellow occurred on a PAD as a result of complexation between Hg(II) and CURNPs. Although a relatively high LOD was observed (0.17 ppm), sensitivity was increased by multiple additions on the PAD to reach an LOD of 0.003 ppm Hg [90].

The oxidation of the 3,3,5,5-tetramethylbenzidine (TMB) by H_2_O_2_ can be catalyzed in the presence of PtNPs, giving rise to a blue color. However, when Hg(II) is present in the medium, this process is inhibited. The detection can be made with the naked eye, yet lower Hg concentrations can be detected after capturing images with a digital camera or smartphone to reach an LOD of 0.01 μM [91].

Upconversion (UC) luminescence allows emission in the visible region after excitation by near-infrared radiation. This strategy shows some advantages, such as decreased photobleaching and autofluorescence interference. Rare-earth-doped b-NaYF_4_ nanoparticles (NPs) have proven to be highly efficient systems for UC luminescence. An upconversion luminescence resonance energy transfer (UC-LRET) sensor was developed by Li and Wang [92] for the detection of Hg(II) in the range of 5 nM to 10 μM in water samples. An LOD of 3.7 nM was obtained. For this, NaYF_4_:15%Yb^3+^,5%Er^3+^ NPs were functionalized with rhodamine B thiolactone (RBT). A hydrogel of this nanocomposite was dropped onto a filter paper. After irradiation with diode laser (980 nm), the green upconversion emission was captured with a digital camera. The sensor showed a great sensitivity and selectivity for Hg in a broad range of pH.

Table 2.: The main applications of PADs for the detection of Hg based on nanomaterials.

**Table 2:** Applications of nanomaterials as receptors in paper-based analytical devices (μ-PADs and paper-based sensors) for the detection of Hg(II).

Material	Type of PAD	Recognition Element	Signal Readout	Sample/Matrix	LOD (ppb)	Ref.
Nitrocellulose membrane	Lateral flow strip *	DNA-conjugated AuNPs	Visual detection	River water Tap water	0.02	[58]
Filter paper	Paper-based sensor	ssDNA-AuNPs	Smartphone	Pond water River water	10	[59]
Nitrocellulose membrane	Lateral flow strip *	Thiol-modified ssDNA- AuNPs	Visual detection and digital camera	Waters	0.005 (naked eye) 0.0015 (quantitative)	[60]
Nitrocellulose	Paper-matrix array *	ssDNA-AuNPs; Signal-enhanced by Ag(I) reduction	Scanner	Tap water Lake water	0.0005 (500 times better after signal amplification)	[61]
Cellulose nanofiber	AuNPs@CNF * membrane	AuNPs	Naked eye	Water	0.2	[62]
Filter paper (salinization)	μ-PAD	N,N0-bis(2-hydroxyethyl) Dithiooxamide-AuNPs	Naked eye and digital camera	Salmon fish and dust storm days	3	[63]
Filter paper	Paper-based sensor	AgNPs, AgNPls	Naked eye and Digital camera	Drinking water Tap water	120 (2 after preconcentration)	[65]
Cellulose	μ-PAD *	AgNPs	Digital camera	Waters	1	[66]
Whatman paper No. 1	μ-PAD *	AgNPs	Smartphone	Waters (tap, pond, etc.)	3 (1 after preconcentration)	[67]
Whatman paper No. 1	Paper-based sensor	Glucose-AgNPs	Digital camera	---	20 (colorimetry) 200 (naked eye)	[68]
Whatman paper No. 1	Paper-based sensor	AgNPs (photosynthesis)	Naked eye and photometry	Aqueous solutions	5 (photometry) 5 × 10^3^ (naked eye)	[69]
Whatman filter	Paper-based sensor *	AgNPs	Smartphone	River water	0.86	[70]
Whatman paper No. 1	Paper-based sensor (CH_3_Hg^+^)	Oxalicalix[4]arene- AgNPs	Colorimetry, fluorimetry, Naked eye	Waters	0.34 CH_3_Hg^+^ (colorimetry) 14 (paper strip)	[71]
Whatman (Different papers)	Paper-based sensor	AgNPs	Smartphone	Waters (River, pond, etc.)	10	[72]
Filter paper	Paper-based sensor	AgNPs	Smartphone	Waters	5.6 (in solution) 60 (PAD)	[73]
Filter paper	Paper-based sensor	CdSe/ZnS and TMB	Smartphone and naked eye	Cosmetic cream Tap water	ca. 18	[78]
Filter paper	Paper-based sensor	MS_2_ QDs	Naked eye under UV lamp	Waters	0.36 (fluorimetry) 2 (naked eye)	[79]
Filter paper	3D Origami m-PAD	Grafted CdTe QDs (Paper@QDs@IIPs)	Fluorescence	Lake water, Seawater	0.056	[80]
Filter paper	Paper strip	N,S-codoped GQDs	Digital camera	Wastewater	0.028 (fluorimetry)	[81]
Carboxylated Cellulose spheres	Fluorescent spherical sponge cellulose (Cu, Hg, Al)	CDs	Digital and mobile cameras after UV irradiation	Tap water River water	5.2 (Hg)	[82]
Filter paper	μ-PAD (Hg, Cu)	N-doped CDs	Digital camera (UV irradiation)	Tap water	1.24 Hg (fluorimetry) 20 Hg (m-PAD)	[83]
Filter paper	Paper-based sensor	CDs	Naked eye (UV irradiation)	Tap water Lake water	0.028 (fluorimetry)	[84]
Filter paper (Different types)	Distance-based μ-PAD	N-doped CDs	Naked eye (UV irradiation)	Drinking water tap water, pond water	5 (after preconc.)	[85]
Filter paper	Paper-based sensor (UV LED irradiation)	N-doped CDs	Smartphone	---	2.14	[86]
Whatman No. 1 paper	μ-PAD (Hg, Ag, antibiotics)	Fluorescent ssDNA- functionalized graphene oxide	Scanner	Spiked water	24.2 (Hg)	[87]
Filter paper	Paper-based sensor *	BSA-AuNCs (conjugated with Pyridoxal)	Naked eye	Fish, tap water, River water	6.38 (fluorimetry) 0.2 (naked eye)	[88]
Filter paper	Paper-based sensor	BSA-AuNCs and Tb(III) (reference)	Naked eye (UV irradiation)	Biological samples	0.2 (fluorimetry) 20 (naked eye)	[89]
Whatman paper No. 1	Paper-based sensor	Curcumin NPs	Digital camera	Waters (several types)	170 (direct) 3 (Prec.)	[90]
Filter paper	Paper-based sensor *	PtNPs and TMB	Digital camera and Smartphone	Pond water Tap water	2	[91]
Filter paper	Paper-based sensor	NaYF4:Yb^3+^/Er^3+^ NPs Functionalized With RBT	Digital camera after diode laser irradiation (980 nm)		0.74 (in solution)	[92]
Filter paper	Paper-based sensor	fluorescent nanoaggregates (FNAs)	Naked eye (UV irradiation)	Tap water, pond water seawater	4 (Hg^2+^) 18 (CH_3_Hg^+^) (fluorimetry)	[93]

* PADs providing an LOD equal or below the maximum contaminant level fixed by US EPA.

## 5. Hg Speciation Using PADs

So far, strategies for the speciation of Hg^2+^ and CH_3_Hg^+^ using PADs have been very scarce [71,92]. Oxacalix[4]arene-templated silver nanoparticles modified with rhodamine B to induce fluorescence as a sensor for the detection of CH_3_Hg^+^ was attempted [71]. Three platforms were employed for the immobilization of the receptor, i.e., cellulose strip, zeolite imidazole framework and alginate beads. For discrimination between Hg^2+^ and CH_3_Hg^+^, an addition of EDTA was needed, which acts as a masking agent toward Hg^2+^.

Fluorescent nanoaggregates (FNAs) on phenanthroline-based amphiphiles, which are sensitive to Hg^2+^ and CH_3_Hg^+^ [93]. A paper-based sensor was built for on-site detection. Fluorescence quenching was caused by both species, but it was higher for Hg(II) and, moreover, CH_3_Hg^+^ required more time (ca. 10 min) to provide a response. Thiolated aminoacids were seen to bind the metal ion center to form a tertiary complex. This facilitates the charge transfer interaction and destabilizes the nanoaggregates. The LODs for both Hg(II) and CH_3_Hg^+^ were 4 and 18 ppb, respectively.

## 6. Conclusions and Outlook

In this overview, state-of-the-art PADs for Hg monitoring in environmental waters have been outlined. With some exceptions, most PADs do not reach the sensitivity required to cope with the Hg levels imposed by the main regulations on drinking water monitoring. For many μ-PADs, the LODs reported are mostly in the ppm region, while a detection ability at the ppb level at least should be required for suitable monitoring of Hg(II) in waters. This drawback can be alleviated when nanomaterials are implemented as receptors in PADs instead of conventional chromogenic/fluorogenic reagents, yet LODs at the ppb level are reported in many cases when nanomaterial-based assays are carried out in a solution format in combination with benchtop analytical instruments (e.g., UV-vis spectrophotometer, fluorimeter).

So far, most PADs developed for Hg(II) detection have been tested directly with synthetic solutions or spiked water samples. However, other, more complex samples typically require some sample pretreatment (e.g., extraction, digestion, masking, etc.) to yield Hg(II) ions in solutions.

Other shortcomings may also arise when PADs based on nanoparticle receptors are applied to real samples due to matrix effects. Thus, capping agents and recognition elements attached to the NP surface can be greatly affected by the high ionic strength and extreme pH values present in many samples. Thus, the main weakness of nanomaterial-based sensors is their limited stability to tackle Hg detection in complex real samples, e.g., seawater, wastewater, biological matrices, etc. As an example, those PADs based on aggregation phenomena of plasmonic NPs are prone to instability, which makes their application troublesome.

In addition, PADs reported for Hg(II) detection are intended for single use and very few publications include studies to evaluate their potential reversibility. The time to reach readout with PADs is highly variable. Thus, the analytical response is commonly achieved almost immediately with PADs, even though non-negligible pretreatment or incubation times can be required for optimal response, thus leading to longer analysis times [51,59,64]. In addition, analysis times can be substantially increased (e.g., 2–12.5 h [65,68,90]) when enrichment steps are required to achieve adequate sensitivity. Among others, headspace sampling, filtration, solid-phase extraction and dropwise addition and evaporation have been reported for the determination of Hg species. In any case, realistic analysis times considering not only the time required for obtaining a noticeable response but also to perform the measurement (e.g., with flatbed scanners after an undefined drying time) should be clearly provided in future works.

Particular attention should also be paid to selectivity studies. In fact, nonrealistic concentration levels of potential interferences are considered in several studies (e.g., 1- to 10-fold interferent-to-Hg species ratio). The tolerance of PADs to potential interferences should be studied in sufficient detail to clearly identify the applicability of developed PADs for the analysis of real samples.

New advances are also needed so as to tackle the main challenges posed by Hg monitoring in the different environmental compartments. The detection of methyl-mercury in environmental samples is of paramount importance, given the enhanced accumulation ability and toxicity displayed by this Hg species, so more attention should be paid to the design of PADs for Hg speciation. Improvements can be expected in next years in the design of more stable, selective and sensitive recognition elements anchored onto cellulose scaffolds as well as more efficient strategies for the preconcentration of Hg integrated with PADs.

## Figures and Tables

**Table 1:** Applications of chromogenic/fluorogenic reagents as receptors in PADs (μ-PADs and paper-based sensors) for the detection of Hg(II).

Material	Type of PAD	Recognition Element	Signal Readout	Sample/Matrix	LOD (ppb)	Ref.
3 MM Whatman chromatography paper	Paper-based sensor	CuI	Scanner	Fish	7 (ng/g)	[41]
Cellulose	Paper-based sensor	bis(ferrocenyl) azine	Naked eye	Aqueous media	104	[42]
Porous silica matrix onto cellulose	Paper-based sensor *	Rhodamine B thiolactone	Flatbed scanner and naked eye	Water	0.24 (Scanner)	[43]
Filter paper	Paper-based sensor	Rhodamine appended vinyl ether	Naked eye	Drinking water Tap water	27.2 (in solution) 10^4^ (paper strip)	[44]
Whatman paper	Paper-based sensor	Ir complex (Phosphorescent)	Naked eye	---	3.56 (fluorimetry)	[45]
Cellulose paper	Paper-based sensor (Hg, I, Zn)	Calix[4]arene (fluorescent)	Digital camera (UV irradiation)	Wastewater	0.58 (fluorimetry)	[46]
Filter paper	Paper-based sensor	Tetrahydrophenazine-based Fluorophore	Digital camera	---	8 × 10^3^ (neutral pH) 3 × 10^3^ (pH 1.6–2.3)	[47]
Filter paper	μ-PAD	Dithizone	Naked eye	Whitening cream	930	[48]
Whatman No. 4 filter paper	μ-PAD	HgI_4_^2−^ complex	Digital camera	Contaminated soil and water	2 × 10^4^	[49]
Whatman grades No. 1 and 4	μ-PAD (Hg, Pb, Cr, Cu, Fe)	Three indicators (ligands)	Digital camera	Waters	20	[50]
Whatman No. 1 paper	μ-PAD (Cu, Co, Ni, Mn, Hg)	Dithizone (for Hg)	Scanner	Drinking, pond and tap water	200 (scanner) *ca.* 10^4^ (naked eye)	[51]
Whatman grade No. 1 filter paper	Array paper strip (for Hg, Ag, Cu)	5 indicators (18 formulations)	Flatbed scanner	Pond water	38 (Hg)	[52]

* PADs providing an LOD equal or below the maximum contaminant level fixed by US EPA.

## Data Availability

No new data were created or analyzed in this study. Data sharing is not applicable to this article.

## Abbreviations

AgNPs	silver nanoparticles
AgNPls	silver nanoplates
AFM	atomic force microscopy
AuNCs	gold nanoclusters
AuNPs	gold nanoparticles
AuNRs	gold nanorods
BSA	bovine serum albumin
BV	biliverdin
CA	citric acid
CCS	carboxylated cellulose spheres
CDs	carbon dots
CHEF	chelation-enhanced fluorescence
CNF	cellulose nanofibrilatted
CQDs	carbon quantum dots
CS-CDs	sponge cellulose fluorescence spherical
CuNPs	copper nanoparticles
CURNPs	curcumin nanoparticles
CV-AAS	cold vapor-atomic absorption spectrometry
CV-AFS	cold vapor-atomic fluorescence spectrometry
DAOC	di-acetamido-oxacalix[4]arene (DAOC)
DNA	deoxyribonucleic acid
EDTA	Ethylenediaminetetraacetic acid
ETAAS	electrothermal atomic absorption spectrometry
FNAs	Fluorescent nanoaggregates
GLU	glucose
GO	graphene oxide
GQD	graphene quantum dots
HEPES	2-[4-(2-hydroxyethel) piperazine-1-yl]ethanesulfonic acid
ICP-MS	inductively coupled plasma-mass spectrometry
ICTs	information and communication technologies
IFP	infrared fluorescence protein
LED	Light emitting diode
LOC	Lab-on-chip
LOD	limit of detection
LRET	luminescence resonance energy transfer
LSPR	localized surface plasmon resonance
MBA	mercaptobenzonic acid
NCs	nanoclusters
NCDs	nitrogen-doped carbon dots
NPs	nanoparticles
PADs	Paper-based analytical devices
μPADs	microfluidic paper-based analytical devices
PET	photoinduced electron transfer
PEI	polyethylenimine
ppb	parts-per-billion
ppm	parts-per-million
PtNPs	platinum nanoparticles
Py	pyridoxal
QDs	quantum dots
RDV	rhodamine appended vinyl ether
RGB	red green blue
SA-LFS	signal-amplified lateral flow strip
SERS	surface-enhanced Raman scattering
SA-LFS	signal-amplified lateral flow strip
SPR	surface plasmon resonance
ssDNA	single-strand DNA
TLm	6-thienyl-lumazine
TMB	3,3,5,5-tetramethylbenzidine
TXRF	total reflection X-ray fluorescence
USEPA	United States Environmental Protection Agency
UC	upconversion
UV	ultraviolet
WHO	World Health Organization
XPS	X-ray photoelectron spectroscopy
